# Dr. Jaques's Case of Poison

**Published:** 1801-06

**Authors:** Tho. Garnett

**Affiliations:** Royal Institution


GENTLEMEN,

I IMAGINE that the following circumſtance, an account of which I have lately received from Dr. Jaques, of Harrowgate, will not be deemed unintereſting or improper for your Journal. I, however, have to regret that the detail is not ſo complete as I could have wiſhed.

Dr. Jaques was deſired to viſit the apprentice of an apothecary at Harrowgate, whoſe head ſeemed very much affected, ſo that he ſcarcely knew either what he did or ſaid, and ſeemed to labour under a kind of inſanity. His tongue was very foul and ſwoln, and he ſpit very much, which induced Dr. J. to ſuſpect that he was under the influence of mercury; this, however, he poſitively denied. As he was very coſtive, Dr. J. preſcribed a laxative a laxative medicine, which operated and relieved his head confiderably. His complaints, however, returned with increafed violence, in confequence of which he was fent home to his relatives, at the diftance of fome miles, where his mafter attended him occafionally.

One day, when his powers of recollection feemed better than ufual, he mentioned a circumftance, which Dr. J. believes was the caufe of his difeafe. A perfon had come to the fhop for fome white arfenic, which he wifhed to have made into pills with butter and wheat flour; thefe pill the young man rolled in his hands, and faid that he was never well after that day, though it was ten days after this before he was obferved to be ill, and three weeks before he mentioned his fufpicion of the caufe of it. Dr. J. faw him after this at his father's houfe, but he was then in the agonies of death; but from what could be learned from his parents, his lower and upper extremities had for fome time been paralytic, with violent pains in the mufcles, as well as the bowels. Dr. J. fays, that he has no doubt in his own mind as to the caufe of his death. This cafe tends to fhew that arfenic, as well as fome other metallic poifons, may be taken into the fyftem by the abforbents, and produce its baneful effects upon the conftitution. Many of the fymptoms in this cafe refembled thofe produced by the poifon of lead, only they were more violent.

I am, &c.

*Royal Inftitution, May 8th,* 1801. THO. GARNETT.

---

